# Laser-Etched Stretchable Graphene–Polymer Composite Array for Sensitive Strain and Viscosity Sensors

**DOI:** 10.1007/s40820-019-0333-6

**Published:** 2019-11-16

**Authors:** Yuting Jiang, Yang Wang, Heting Wu, Yuanhao Wang, Renyun Zhang, Håkan Olin, Ya Yang

**Affiliations:** 1 CAS Center for Excellence in Nanoscience, Beijing Key Laboratory of Micro-nano Energy and Sensor, Beijing Institute of Nanoenergy and Nanosystems, Chinese Academy of Sciences, Beijing, 100083 People’s Republic of China; 2 Xinjiang Technical Institute of Physics and Chemistry, Chinese Academy of Sciences, Urumqi, Xinjiang, 830011 People’s Republic of China; 3 Department of Natural Sciences, Mid Sweden University, Holmgatan 10, 85170 Sundsvall, Sweden; 4 School of Nanoscience and Technology, University of Chinese Academy of Sciences, Beijing, 100049 People’s Republic of China; 5 Center on Nanoenergy Research, School of Physical Science and Technology, Guangxi University, Nanning, 530004 People’s Republic of China

**Keywords:** Hydrophobic smart coatings, Flexible sensors, Soft materials, Controlled drops, Graphene

## Abstract

The ability to control surface wettability and liquid spreading on textured surfaces is of interest for extensive applications. Soft materials have prominent advantages for producing the smart coatings with multiple functions for strain sensing. Here, we report a simple method to prepare flexible hydrophobic smart coatings using graphene–polymer films. Arrays of individual patterns in the films were created by laser engraving and controlled the contact angle of small drops by pinning the contact lines in a horizontal tensile range of 0–200%. By means of experiments and model, we demonstrate that the ductility of drops is relied on the height-to-spacing ratio of the individual pattern and the intrinsic contact angle. Moreover, the change of drop size was utilized to measure the applied strain and liquid viscosity, enabling a strain sensitivity as high as 1068 μm^2^/%. The proposed laser-etched stretchable graphene–polymer composite has potential applications in DNA microarrays, biological assays, soft robots, and so on.

**Electronic supplementary material:**

The online version of this article (10.1007/s40820-019-0333-6) contains supplementary material, which is available to authorized users.

## Highlights

- A simple method to prepare flexible hydrophobic smart coatings was proposed.
- The change of drop size was utilized to measure the applied strain and liquid viscosity.
- The prepared composite film exhibits favorable stretchability, high flexibility, and outstanding ability of controlling the drop shape.

**Electronic supplementary material:**

The online version of this article (10.1007/s40820-019-0333-6) contains supplementary material, which is available to authorized users.

## Graphical abstract

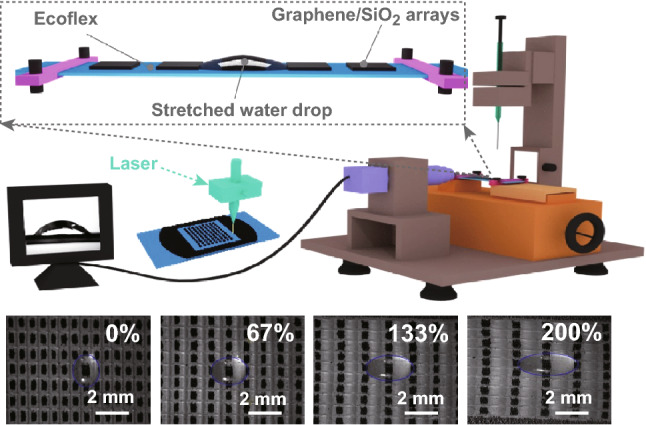

## Introduction

Repellent smart coatings, which can regulate surface wettability and control drops by interacting with the external stimulus or adopting the specific design of hierarchical textures, are significant in a great variety of applications ranging from digital lab on a chip, drag reduction, separations to tactile sensing [1–7]. Various attempts have been implemented to achieve smart coatings. For example, Jiang and co-workers demonstrated a TiO_2_ nanorod film with switchable surface wettability, which can be transformed between superhydrophilicity and superhydrophobicity by using UV light [8]. Wang et al. [9] utilized asymmetric nanostructured surfaces to realize controllable liquid spreading. Recently, the increasing complicacy of scientific research and industrial production has demanded innovative smart coatings that can combine stretchable and hydrophobic functionalities to adapt flexible elements. Such coatings should possess three significant characteristics: high extensibility, variable surface wettability, and versatility in the application.

Creating flexible smart coatings is a significant challenge, because it demands a favorable integration of flexible substrates and repellent materials. Soft materials, such as elastomers, paper, and hydrogels, offer exciting promise [10–20]. For example, Jiang et al. [21] produced superamphiphobic paper by two-step method. Zhao et al. [22] demonstrated a smart coating with tunable wetting by elastic topologically grooved poly(dimethylsiloxane) films. Among these materials, silicone rubber (Ecoflex) films used as substrates for flexible devices have obtained extensive attention because of their high stretchability and admirable accessibility. The surface wettability of the coating is regulated by a series of characteristics including surface structures and surface chemistry [23–26]. Recently, graphene and carbon nanotubes have been chiefly used because they exhibit inherent hydrophobicity, high conductivity, and surface texture, even under mechanical strain [27–30]. For example, Zhao et al. [31] developed a smart coating with the tunable wettability by using porous graphene films. Mates et al. [32] presented a stretchable film with stable superhydrophobicity and the restorable electrical feature by combining Parafilm-M and carbon nanofibers. Although many researches provided advancements in flexible hydrophobic coatings, few studies presented smart coatings with flexibility for strain sensing by dynamically controlling the shape and contact angle of the droplets.

Here, we report a method to prepare flexible hydrophobic smart coatings that can control the contact angles of small water drops in a horizontal tensile range of 0–200%. The coatings can be achieved by filtering graphene/SiO_2_ suspension and peeling off the solidified silicone rubber (Ecoflex) films. In the composite films, we utilized laser engraving method to produce arrays of individual patterns, which were availably used to control the contact angles of the drops by pinning the contact lines. The coatings are stretchable and hydrophobic and enable a strain sensitivity by measuring the change of the drops in a horizontal tensile range. These properties make the laser-etched stretchable graphene–polymer composite films for a drop regulation in various fields.

## Experimental Section

### Materials and Methods

Graphene (~ 20 μm thick), hexadecyl trimethyl ammonium bromide (CTAB), and silica particles (diameter of ~ 5 μm) were purchased from Aladdin (purity > 99.99%). Silicone elastomer (Ecoflex 0020) was purchased from Smooth-On. Hydroxypropyl distarch phosphate, sheep blood, and phosphate-buffered saline solution (PBS) were purchased from Wuhan Chundu Biology Company.

CTAB and graphene were mixed at a ratio of 1:2.5 by weight with silica particles as follows: Graphene (0.25 g) and different amounts of silica particles were added into the CTAB (0.75 g) solution in 100 mL deionized water. Then, the obtained solution was kept sonicating for 1.5 h to allow the homogeneous dispersion of graphene and silica particle, followed by a setting time of 1 h to assure the sediment of the impurities included. Finally, the mixed solution was transferred to the container, leaving out the impurities. To make the composite film, a certain amount of the solution diluted by deionized water was filtrated in the process of vacuum filtration and washed by water and ethyl alcohol alternatively for three times, with the composite film attached on the filter paper obtained. Then, the filter paper was dried at 60 °C for 5 min. The dried paper was put into a mold, followed by the addition of Ecoflex solution which was the mixture of Part A and Part B by a ratio of 1:1 by weight. After being vacuumized, the above mold was kept at 80 °C for 1 h to let Ecoflex solution cure. Then, the filter paper was removed with the composites on the cured Ecoflex film. Finally, the composite arrays (12.8 × 13 mm^2^) were obtained by a laser cutting machine. The graphene/Ecoflex composite film and SiO_2_/Ecoflex composite film were prepared in the same way. A composite film in a larger size (12.8 × 38.8 mm^2^) was also made by the above procedures for the measurement of angle. To obtain sheep blood of different viscosities, the bought blood was diluted and thickened by PBS solution and hydroxypropyl distarch phosphate, respectively.

### Characterization and Measurements

The structure and morphology of the composite film and the materials used were characterized by scanning electron microscopy (SEM, SU8020). And the sizes of the arrays on the film were measured by optical microscope (NiKon, DS-Ri2). The viscosities of different liquids were measured by a viscometer (NDJ-79). The droplet of about 8 μL was produced from a stainless steel needle. The strains applied on the film were created by a stretching device, which was put in front of the camera of a contact angle meter (SCI3000F). Once a strain was applied, the picture of the corresponding water contact angle was captured by the computer and analyzed later by a contact angle software program. The top views of the liquid drop at different strains were obtained by rotating the contact angle meter by 90° with the camera above the liquid drop. And then, areas of the measured liquid drop from the top view were obtained by an analysis software program (Imagine J). A fixture was also made to fix the film and create different angles on it.

## Results and Discussion

**Fig. 1:**
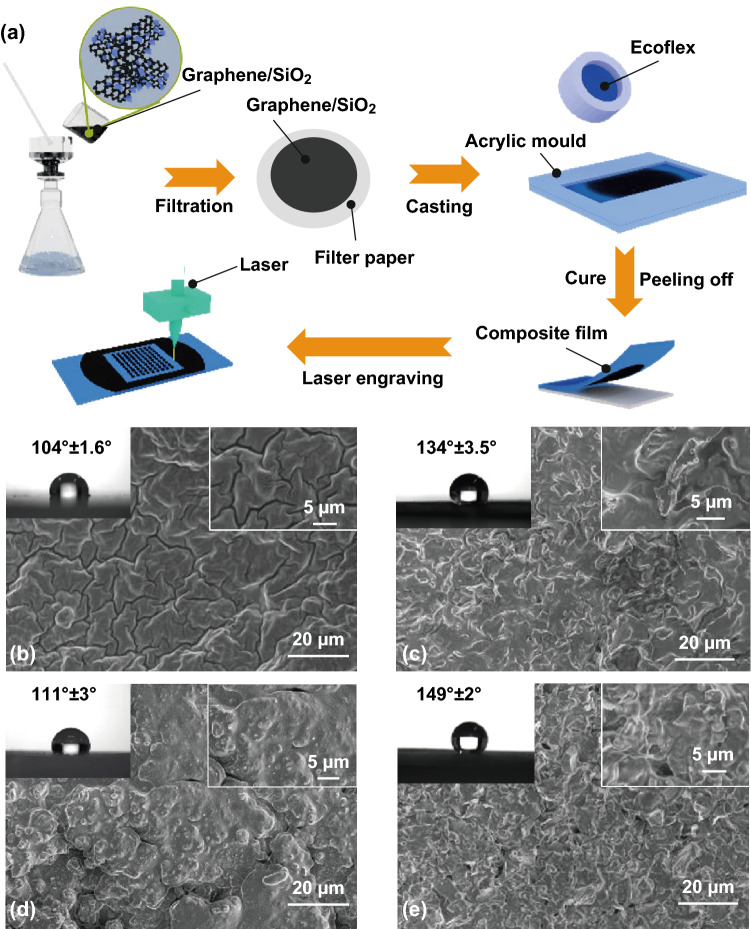
**a** Schematic diagram of the preparation of the composite with arrays. SEM images of **b** Ecoflex film, **c** graphene/Ecoflex composite film, **d** SiO_2_/Ecoflex composite film, and **e** graphene/SiO_2_/Ecoflex composite film. The insets in **b**–**e** are the corresponding water contact angles

The manufacturing procedure of the graphene–polymer composite films is demonstrated in Fig. 1a. As a first step, we fabricated graphene/SiO_2_ composite coatings on the filter paper using filtration method. These coatings present favorable hydrophobicity because graphene possesses an inherent repellent feature, and nano-sized SiO_2_ particles (diameter of ~ 5 μm) can increase surface roughness, which could affect surface wettability. The filter paper was fixed by the acrylic plates, and silicon rubber was cast on the surface. The thickness of rubber film was about 1.5 mm. When the rubber (Ecoflex) film was fully cured, it was peeled off and arrays of individual patterns in the films were created using laser engraving. The influence of the hydrophobic performance on the composite film was investigated. Figure 1b displays that the pure rubber film with some cracks has a water contact angle (WCA) of about 104°. It is generally known that surface energy and surface roughness can affect the wettability. After coating the rubber film with the graphene coating, we found that some wrinkle structures appeared on the film, and WCA was increased to about 134° because the graphene coating possesses lower surface energy than that of silicone rubber (Fig. 1c). When we added SiO_2_ particles into the rubber film, the surface roughness was obviously increased and WCA was about 111° (Fig. 1d). After coating the rubber film with graphene/SiO_2_ coatings, the composite film with bulge structures achieved a high WCA of about 149° (Fig. 1e). The coating thickness is about 30 μm, as shown in Fig. S1a. The WCA was measured as a function of graphene/SiO_2_ mass fraction, where the optimized value is indicated (Fig. S2). The WCA on the composite film increased with the increasing ratio of SiO_2_ particles. When the mass fraction is 4/1, the WCA achieved the maximum value of 149°. Subsequently, the WCA descended because massive SiO_2_ particles could destroy the continuity and hydrophobicity of the graphene coating.

**Fig. 2:**
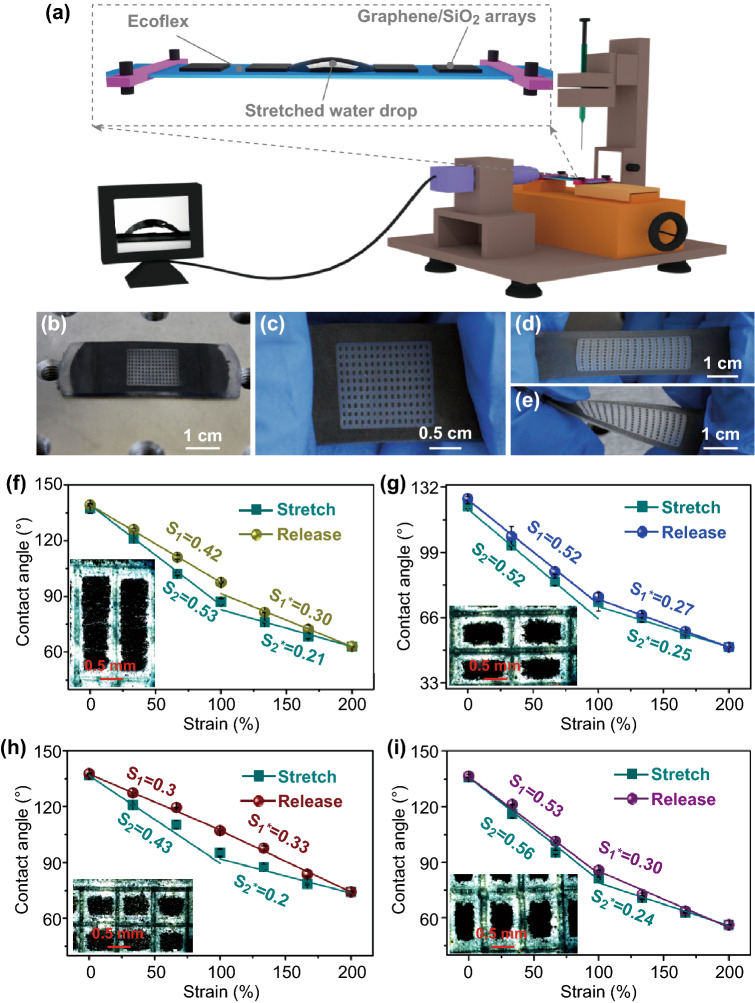
**a** Schematic diagram of the measurement device for water contact angles of the tested films at different strains. **b**–**e** Photographs of the laser-etched graphene/SiO_2_/Ecoflex composite films stretched by different strains. **f**–**i** Water contact angle–strain curves for the composites film with arrays of 15 × 5, 10 × 15, 15 × 15, and 15 × 10. The insets in **f**–**i** are the corresponding optical micrographs of the films

The schematic diagram of the test equipment for the change of the drops and the strain sensing performance is illustrated in Fig. 2a. The prepared films were fixed on the sliding rail, which was used to control the tensile strain of the films. When the drops were deposited on the film, its shape is symmetric, which is determined by a minimization of total surface energy (Fig. 1e). The change of the contact angle on different strained films was observed by a contact angle meter. When we, respectively, brought pulling force to the pure silicone rubber film, the graphene composite film, and the graphene/SiO_2_ composite film, the contact angle of the drops on this film will reduce with the applied strain. However, we found that this process is unstable, and the drops will shrink toward the center, leading to increase the contact angle (Fig. S3b, c). As we pulled the film, the balance was broke, and shape of the drops will be stretched due to the capillary force and static friction. But, the drops will constantly shrink to form a new balance (Fig. S3a and Movie-S1). Previous studies indicated that the lateral adhesion force between the drops and the substrate was determined by the surface tension, the WCA, and the width of the drops [33]. To increase the adhesion force, the arrays of individual patterns were prepared in the films by using laser engraving as illustrated in Fig. 2b, c. The space between two patterns is about 40 μm, and the etched depth is about 70 μm (Fig. S1b). The prepared film possesses an excellent stretchability and a robust feature, as displayed in Fig. 2d, e. Although the obvious sliding of the water drop on the patterned pure rubber film (Movie-S2) can still be observed, we found that the prepared patterns can effectively prevent drops to slide on the graphene/SiO_2_ composite film (Fig. S4 and Movie-S3). Moreover, the change of the contact angle with respect to applied tensile strain can be regulated (Fig. S5). The strain sensitivities (*S*) defined as the slope of curves (*δ*(*C* − *C*_0_)/*C*_0_)/*δ*_ɛ_) are presented in Fig. 2f–i.

**Fig. 3:**
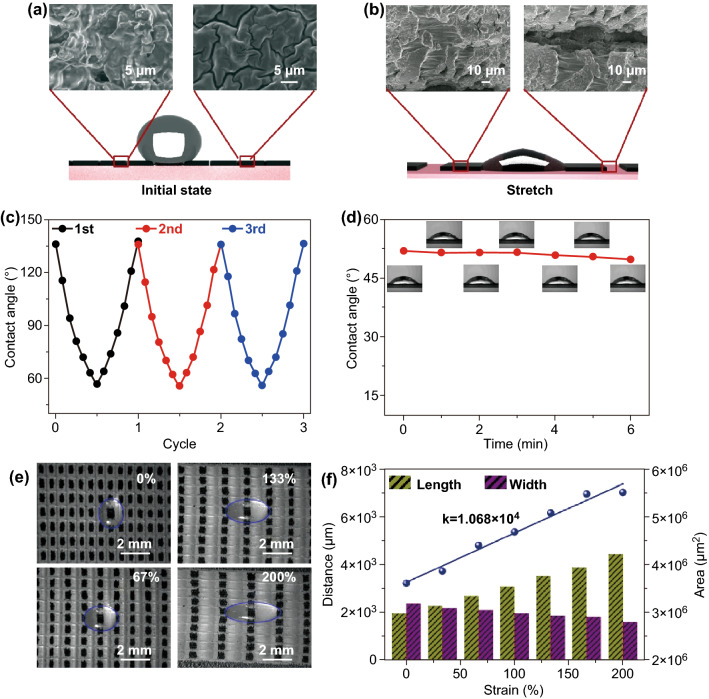
**a**, **b** SEM images of the arrays and interspace between on the film and the schematic diagrams of the composite film at the initial state and stretching state, along with the corresponding water contact angles and optical photographs. **c**, **d** Cycle performance stability performance of the composite film with arrays of 10 × 15. **e** Photographs of the stretched water drop from the top view at different strains. **f** The distance–strain and area–strain curves of the composite film

To explicate the above phenomena, we measured the surface topography at different states. At the start state, the composite film is superhydrophobic because the graphene covered the surface perfectly (Fig. 3a). When the film was stretched to the tensile strain of 200%, massive cracks emerged on the graphene film, and the exposed rubber film could reduce the hydrophobicity of the composite film (Fig. 3b). In addition, the width of the channel between two patterns was magnified. The edge of the drops will pin in the channel due to capillary force generated by these channels. Consequently, the capillary force could enhance the threshold force between the drops and the film and prevent drops to slide on the film, enabling the change of the contact angle along with the film stretching (Fig. S6). Figure 3c illustrates the change of the contact angle of the composite film during periodic stretching in the tensile range of 100–200%. Figure 3d presents the stability of the drops at the tensile range of 200%. Due to the evaporation of liquid, the volume of drops reduced after about 4 min, leading to tiny defects.

In addition to the strain sensitivities, the area and horizontal length of the drops also serve as strain sensing performances, due to their high sensitivity. Changes in arrays of individual patterns caused the size of the drops to vary, allowing the area and horizontal length of the drops to sense tensile strain. From a top view, the drops stood on the middle of carven graphene pattern at the tensile strain of 0% (Fig. 3e). With increasing the strain, the horizontal length of the drops increased, and longitudinal length reduced because the edge of the drops pinned in the channel between two carven graphene patterns, preventing it to slide along the horizontal direction. Meanwhile, the width of the channel in the film enlarged, and larger area of drops spreads over the surface of exposed rubber film. To analyze these variations, we utilized threshold algorithm, which can convert an image to a binary image, to calculate the length and area of the drops at different tensile strains. The variable tendency of the length and area of the drops has been calculated (Fig. 3f). The strain sensitivities (*S*) defined as the slope of curves were 1068 μm^2^/%, showing an outstanding performance. The tendency was also characterized on the flat rubber film and graphene composite film without arrays of individual patterns (Fig. S7). There are negligible changes for both length and width of the drops on these films. The results further indicate that arrays of individual patterns are important to prevent drops to slide on the flexible film. To enhance the variable range of the contact angle, surface compositions are also significant. For example, the pure rubber film with the arrays can achieve the function to control the drops steadily, but the change of contact angle is small due to the lack of the graphene/SiO_2_ composite coatings (Fig. S8). For a larger tensile strain (for example 267%), the drops on the composite film were observed to slide on the film, where the drops shrank to the center continually, and its length and area reduced (Fig. S9). This result can be attributed to the reduction in capillary force. When the composite films were stretched over the tensile strain of about 267%, the bigger gap between graphene/SiO_2_ patterns could not provide adequate capillary force to impede the drop sliding.

**Fig. 4:**
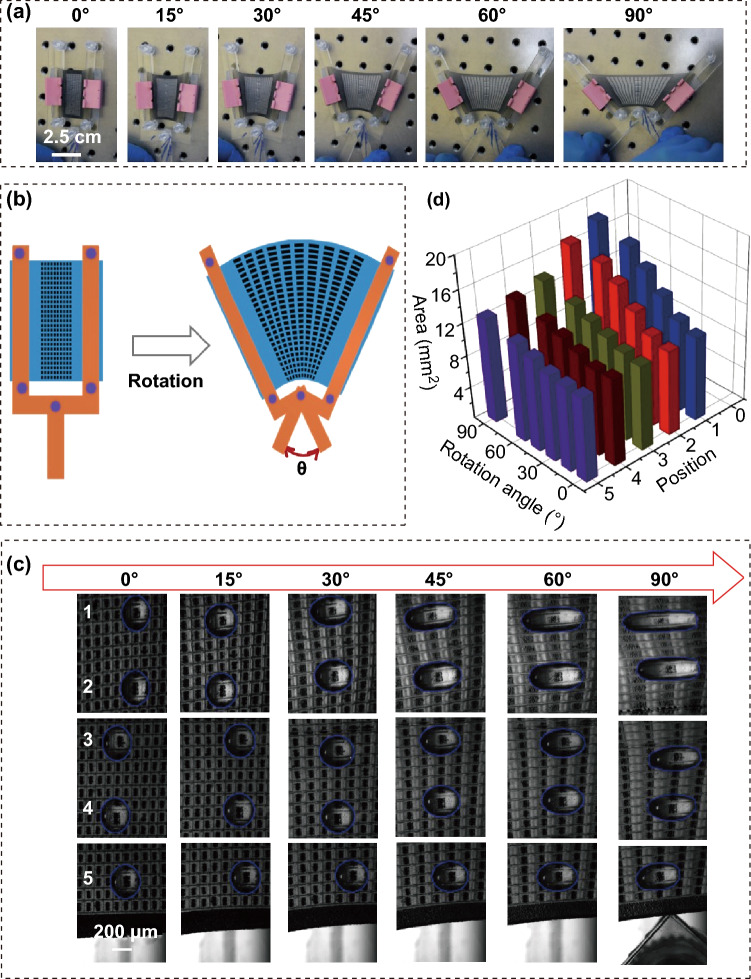
**a** Photographs of the composite film subject to different angles created by a fixture. **b** Schematic diagram of the fixture in original and working states. **c** The bar graph of areas of the stretched water drop versus the applied angles at different positions marked as 1, 2, 3, 4, 5. **d** Photographs of the water drop at different positions and different angles

The prepared composite film exhibits favorable stretchability, high flexibility, and outstanding ability of controlling the drop shape, so it can be used to consider the comprehensive applications. The film was fixed to two fan-shaped fixtures, which can be continuously turned similar to the paper fan, as displayed in Fig. 4a. Five drops were dripped on the centrality of the film in the proper sequence. With the rotation of splints, the film was stretched to fan-shaped states, leading to different shapes of drops on its surface. Figure 4b presents a schematic drawing of the patterns during the rotation. Due to different tensile strains, the deformations of patterns are different on the film, where the outside patterns are bigger than those of the inside patterns. As a result, the stretched length of the drop was affected according to different positions and angles of rotation, as depicted in Fig. 4c. Therefore, these parameters also can be confirmed by measuring the areas of the drops as shown in Fig. 4d, realizing an array of strain sensors.

**Fig. 5:**
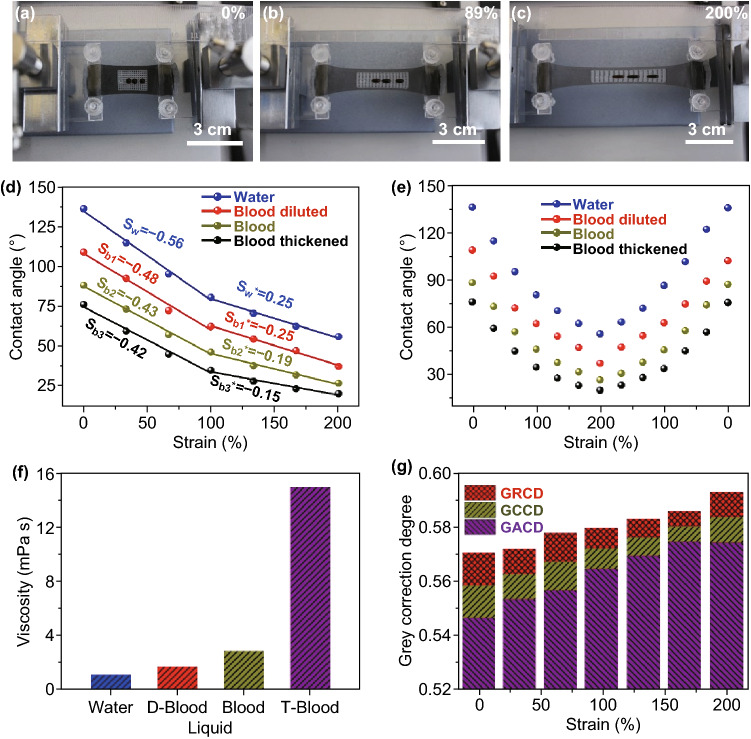
**a**–**c** Photographs of the film with the stretched liquid drop of different viscosities. **d** Curves of contact angle–strain for different liquids. **e** Cycle performance of different liquids. **f** Viscosities of liquids used. **g** Grey relational degree of the film among the applied strains, the corresponding contact angles, and viscosities

Moreover, the composite film can be used to control various liquids with different viscosities. Because the viscosity and the shape of blood are two important parameters in medical domain, we mainly investigated four kinds of liquid: water drops, blood, diluent blood, and thickened blood (blood source from sheep). The corresponding photographs of these drops with different tensile strains are presented in Fig. 5a–c. The results demonstrated that the prepared composite film could effectively regulate the liquids to various shapes by stretching the film (Movies-S4, S5). In addition, the contact angles for these drops under same tensile strain are obviously different and constantly decreased with the increasing tensile strain as shown in Fig. 5d. This process was reversible, as illustrated in Fig. 5e. Moreover, the change of contact angle can also be in turn utilized to discriminate liquid viscosity. Figure 5f displays the viscosity of four drops by using conventional viscosity meter (Fig. S10). To further prove the relativity between the contact angle and liquid viscosity, we used grey relational analysis, which can calculate associated degree based on various factors, to study the corresponding relation. Three parameters were obtained by software (GMS6.0), which consist of relative correlation degree, comprehensive correlation degree, and absolute correlation degree, as depicted in Fig. 5g. All of them were bigger than 0.5, and the results demonstrated that there is a favorable relativity between contact angle and liquid viscosity.

As compared with previous rigid substrates for controlling surface wettability and drop shapes, our composite film possesses excellences in good high flexibility and dynamic control. We used graphene/SiO_2_ coatings to increase surface hydrophobicity and arrays of individual patterns to prevent drop sliding. The preparation process is simple and can be easily realized. For practical application, the change of drop length and contact angle can also be in turn used to estimate the applied strain and angle of rotation and to analyze liquid viscosity. Considering that traditional viscosity analysis is often complex, the composite film provides a novel simple method. Moreover, the film can be used as a strain sensor by measuring the change of the contact angle, averting abundant wires as compared with conventional electric measurements.

## Conclusion

In summary, a flexible composite film, composed of graphene/SiO_2_ composite coatings with arrays of individual patterns and silicone rubber films, was developed for the effective control of the liquid drops, lateral tensile sensing, and analysis of liquid viscosity. The film possesses alterable surface wettability and favorable stability to high tensile strain. Moreover, the multifunctional functions of the composite films will expand the potential applications of other flexible hydrophobic films.

## Electronic supplementary material

Below is the link to the electronic supplementary material.

Supplementary material 1 (PDF 1605 kb)

Supplementary material 2 (MOV 2946 kb)

Supplementary material 3 (MOV 1239 kb)

Supplementary material 4 (MOV 6739 kb)

Supplementary material 5 (MOV 7929 kb)

Supplementary material 6 (MOV 7046 kb)
