# Supplementary material for: Laser-Etched Stretchable Graphene–Polymer Composite Array for Sensitive Strain and Viscosity Sensors
Source: Nanomicro Lett. 2019 Nov 16;11:99. doi: 10.1007/s40820-019-0333-6 (PMC7770859; doi:10.1007/s40820-019-0333-6)
Supplement: Supplementary file 1 — Supplementary material 1 (PDF 1605 kb) [file 40820_2019_333_MOESM1_ESM.pdf]

Supporting Information for

Laser-Etched Stretchable Graphene-Polymer Composite Array for Sensitive Strain and Viscosity Sensors

Yuting Jiang[1, 4, ‡], Yang Wang[1, ‡], Heting Wu[1, 4], Yuanhao Wang[2, *], Renyun Zhang[3], Håkan Olin[3], Ya Yang[1, 4, 5, *]

[1]CAS Center for Excellence in Nanoscience, Beijing Key Laboratory of Micro-nano Energy and Sensor, Beijing Institute of Nanoenergy and Nanosystems, Chinese Academy of Sciences, Beijing 100083, People's Republic of China

[2]Xinjiang Technical Institute of Physics & Chemistry, Chinese Academy of Sciences, Urumqi, Xinjiang 830011, People's Repubic of China

[3]Department of Natural Sciences, Mid Sweden University, Holmgatan 10, SE-85170, Sundsvall, Sweden

[4]School of Nanoscience and Technology, University of Chinese Academy of Sciences, Beijing 100049, People's Repubic of China

[5]Center on Nanoenergy Research, School of Physical Science and Technology, Guangxi University, Nanning 530004, People's Repubic of China

‡ These authors contributed equally to this work.

*Corresponding authors. E-mail: wangyh@ms.xjb.ac.cn (Yuanhao Wang); yayang@binn.cas.cn (Ya Yang)

Supplementary Figures

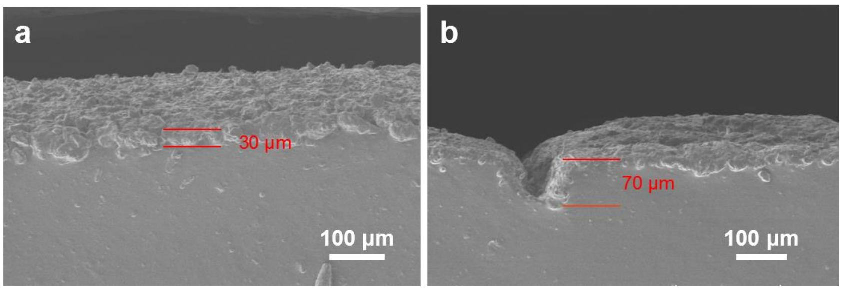

Fig. S1 a Cross-sectional SEM image of the of the Graphene/SiO_2/Ecoflex films. **b** Cross-sectional SEM image of the of the Graphene/SiO_2/Ecoflex films by laser erosion

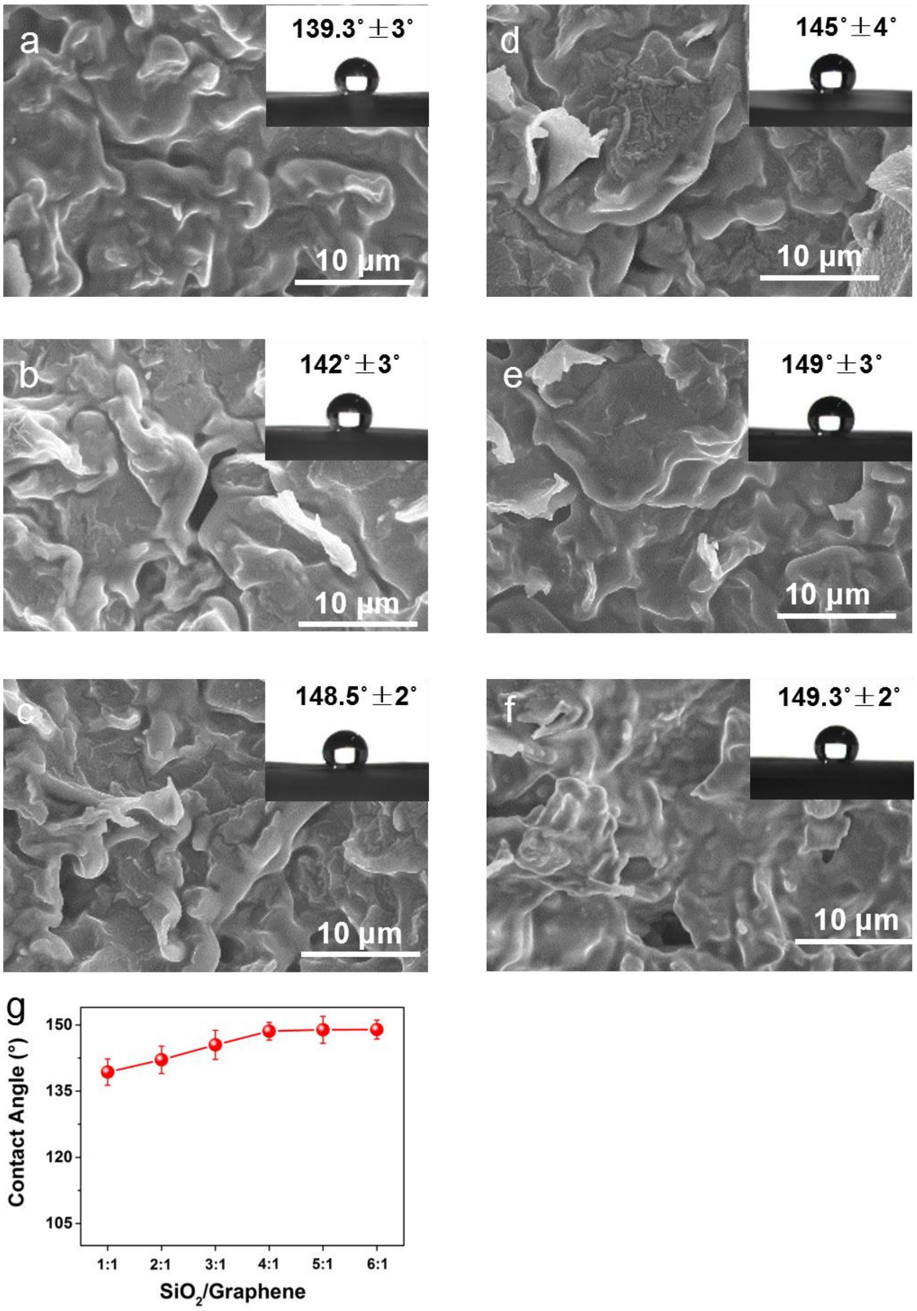

Fig. S2 a-f SEM images of Graphene/SiO_2/Ecoflex films with Graphene and SiO_2 at different rations by weight and the insets are their corresponding water contact angles. **g** Curve of water contact angle versus rations of Graphene and SiO_2 (SiO_2/Graphene=1:1~1:6)

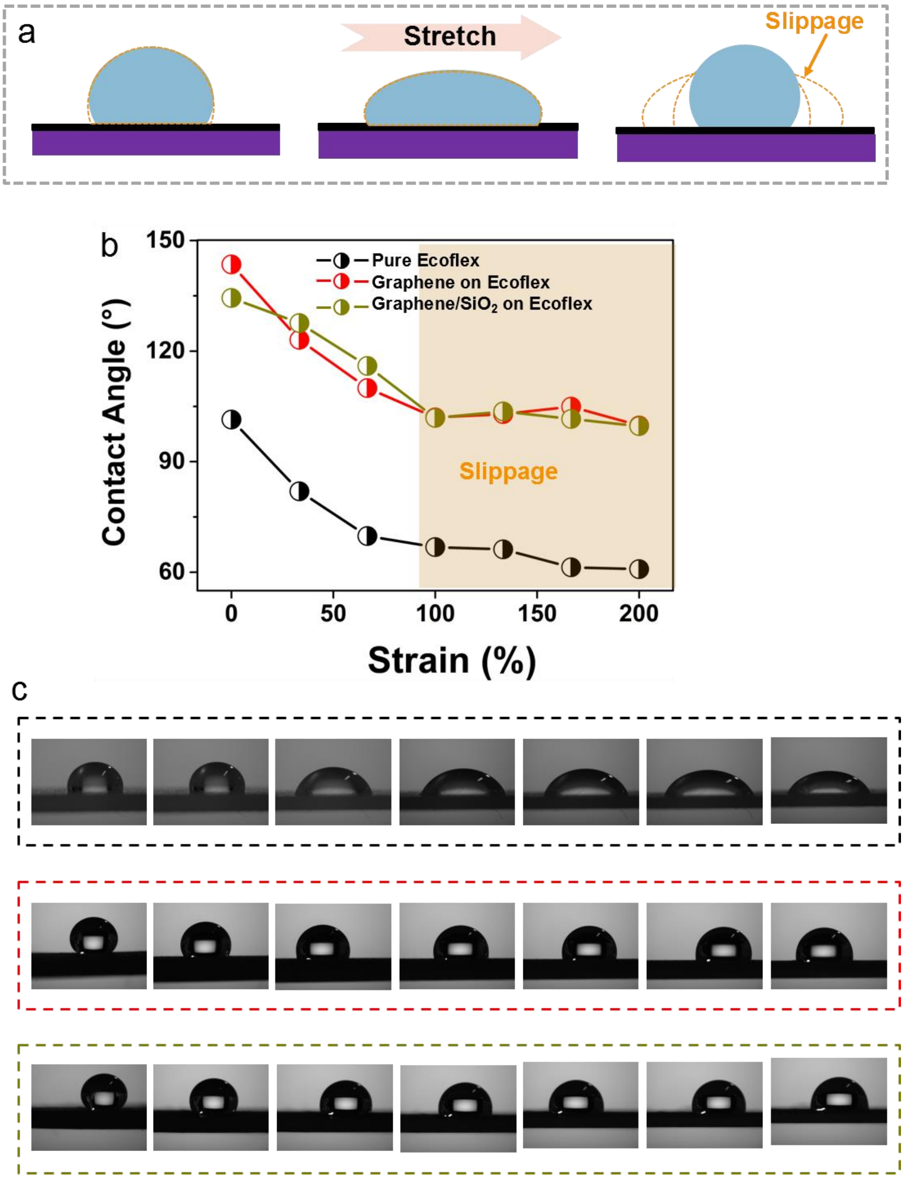

Fig. S3 a Schematic diagram of the film at different strains. **b** Curve of contact angle versus strain for Ecoflex film, SiO_2/Ecoflex and Graphene/ Ecoflex film. **c** Photographs of the stretched water drop for different composite films under the strain from 0% to 200%

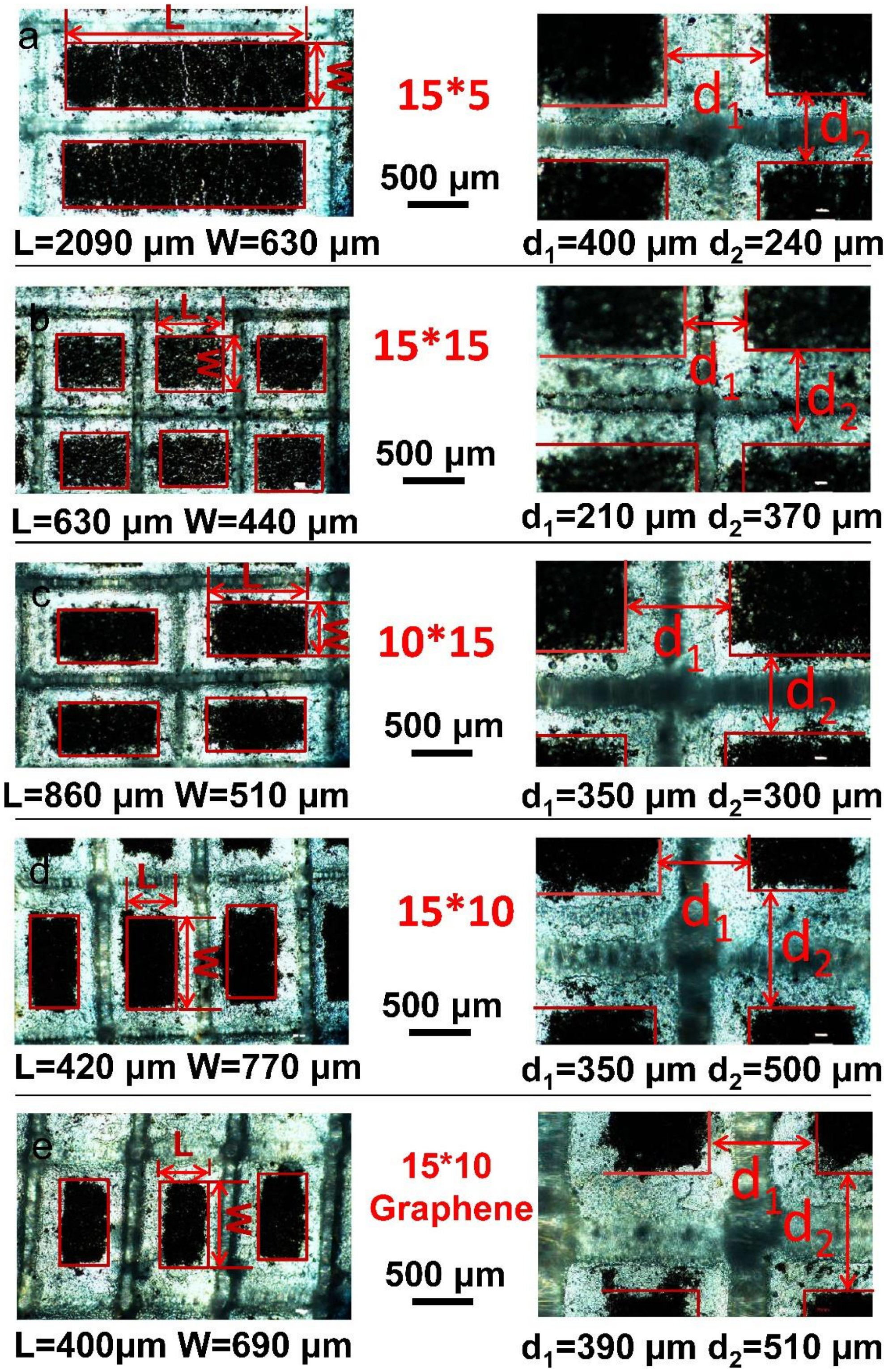

Fig. S4 a-e Optical photographs of Graphene/SiO_2/Ecoflex films with different arrays

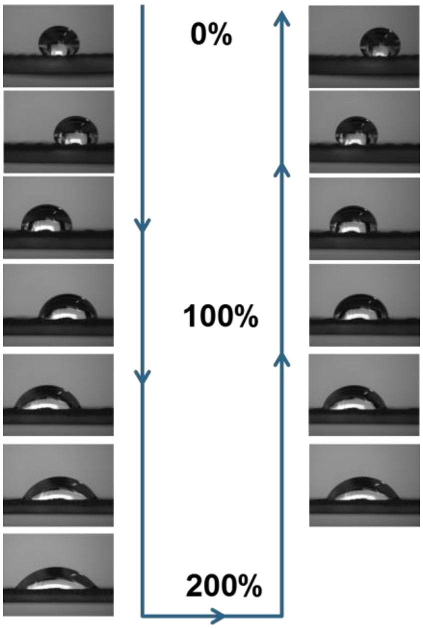

Fig. S5 The change of the contact angle with respect to applied tensile strain

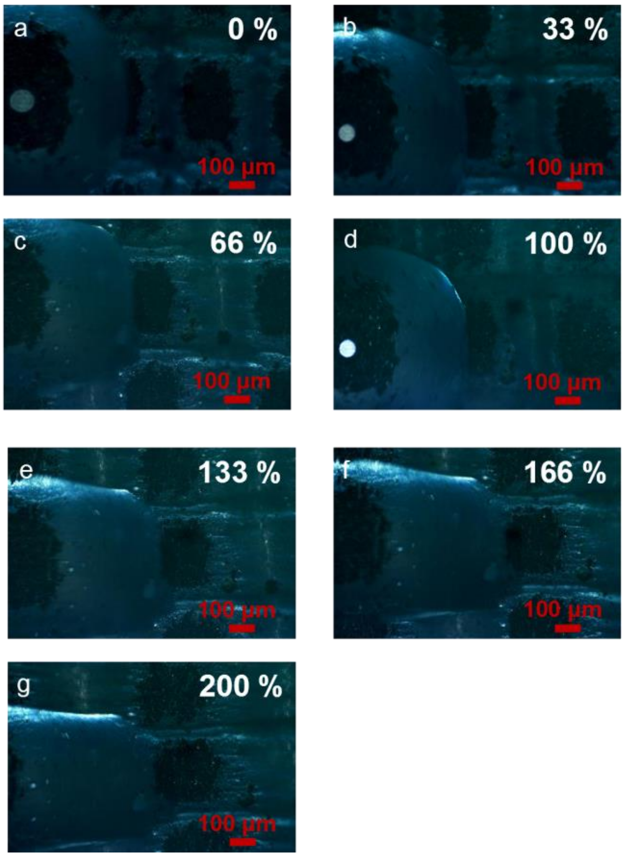

Fig. S6 Change of the contact angle along with the film stretching. **a-g** Optical photographs of the stretched water drop at strains from 0% to 200%

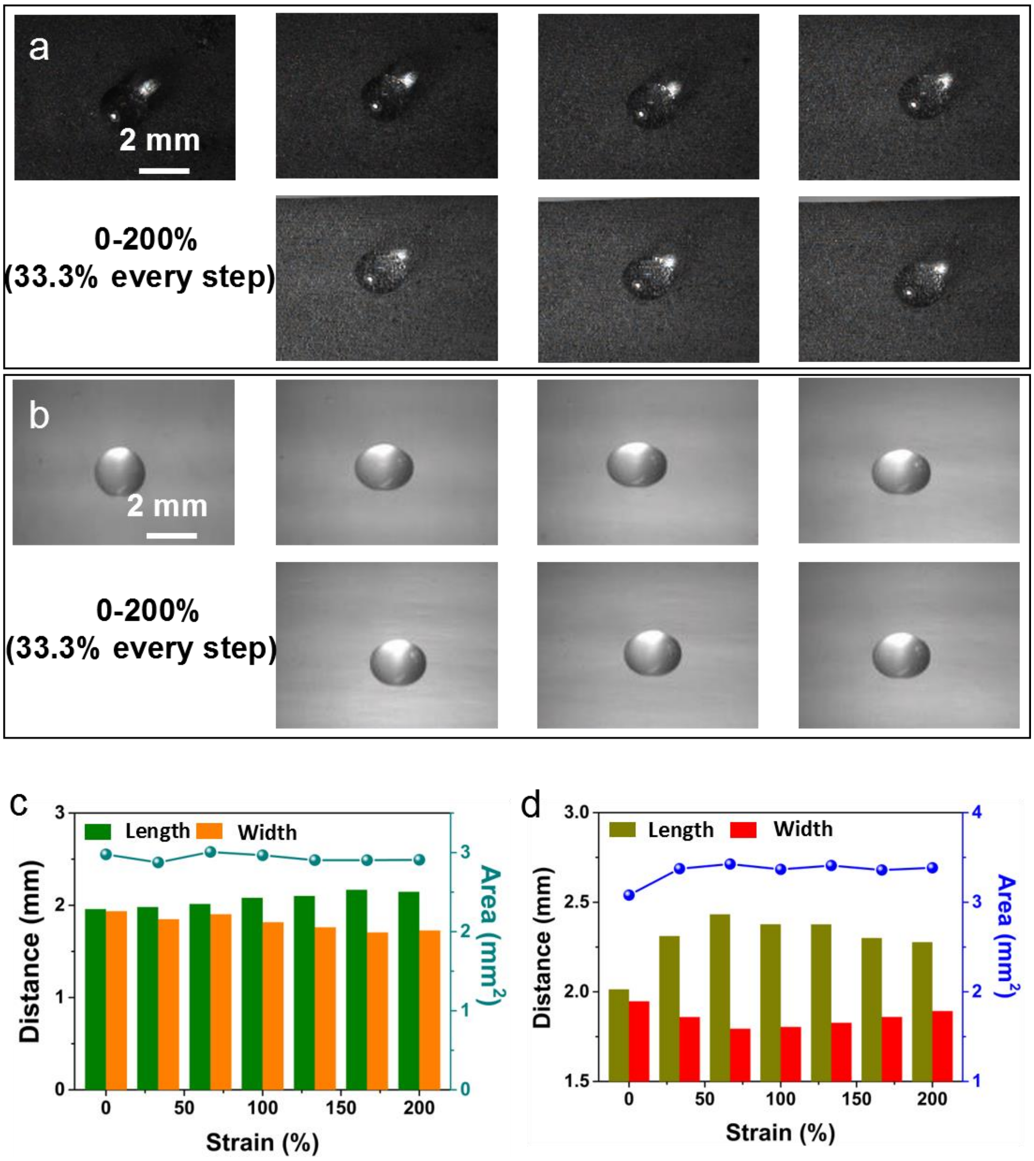

Fig. S7 The change for the flat rubber film and graphene/SiO_2 composite film. **a** Photographs of Graphene/Ecoflex composite film under strains from 0%-200%. **b** Photographs of Ecoflex film under strains from 0%-200%. **c** Curve of area versus strain for Graphene/Ecoflex composite film. **d** Curve of area versus strain for film respectively.

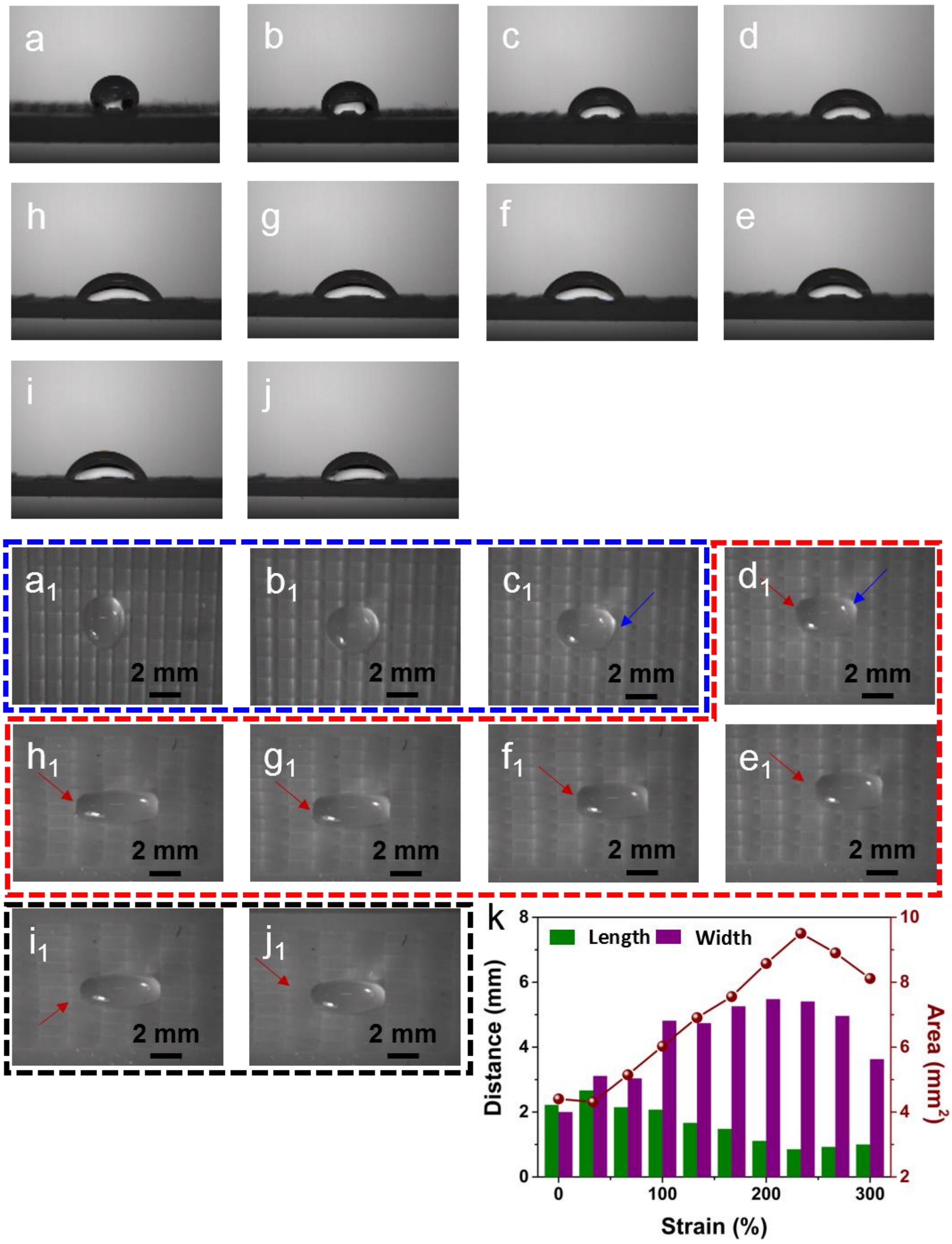

Fig. S8 a Photographs of Graphene/Ecoflex composite film under strains from 0%-200%. **b** Photographs of Ecoflex film under strains from 0%-200%. **a$_1$, b$_1$** Curve of area versus strain for Graphene/Ecoflex composite film and Ecoflex film

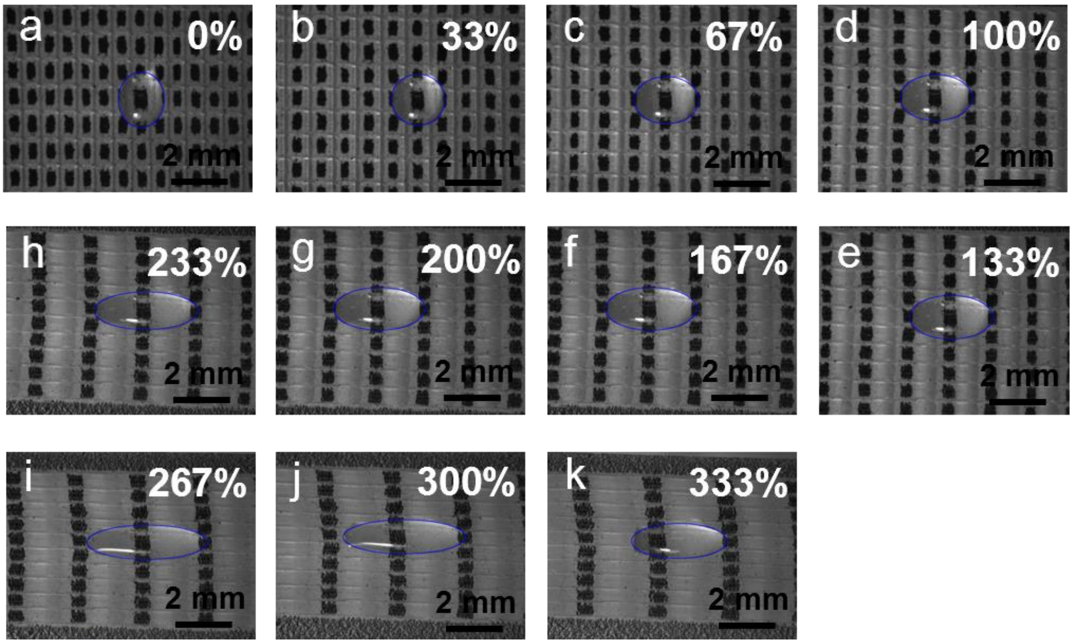

Fig. S9 a-k Photographs of the Graphene/SiO_2/Ecoflex composite film and drop shape under strains from 0%-333%

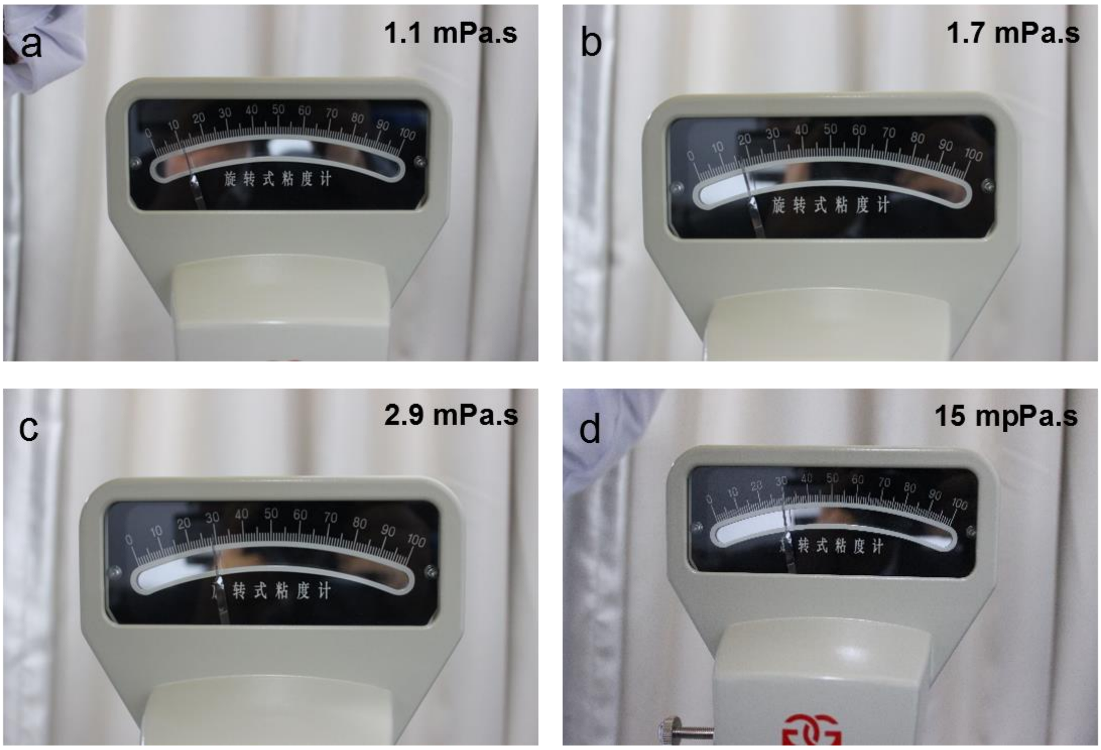

Fig. S10 a-d Photographs of viscosity values for water, blood, diluted blood and thickened blood respectively

Supplementary Movies

Moive-S1 Demonstration of the drop sliding on the pure rubber film

Moive-S2 Demonstration of the drop with still sliding on the pure rubber film with arrays of individual patterns

Moive-S3 Demonstration of the drop without sliding on the composite film with arrays of individual patterns

Moive-S4 Demonstration of blood drops changing with stretching the film

Moive-S5 Demonstration of contact angle of different drops with stretching the film
